# Answers to Correspondents

**Published:** 1909-06-19

**Authors:** 


### Editor's Letter-Box.

### ANSWERS TO CORRESPONDENTS.

"Tabetic" (Darlington).—One of the most complete monographs upon tabes dorsalis (locomotor ataxy) is published by Dr. Byrom Bramwell in his "Clinical Studies," Vol. VI., 1908. The publishers are Messrs. R. and R. Clark, Ltd., Edinburgh.

